# Domestic Dogs as Sentinels for West Nile Virus but not *Aedes*-borne Flaviviruses, Mexico

**DOI:** 10.3201/eid2805.211879

**Published:** 2022-05

**Authors:** Edward Davila, Nadia A. Fernández-Santos, José Guillermo Estrada-Franco, Lihua Wei, Jesús A. Aguilar-Durán, María de J. López-López, Roberto Solís-Hernández, Rosario García-Miranda, Doireyner Daniel Velázquez-Ramírez, Jasiel Torres-Romero, Susana Arellano Chávez, Raúl Cruz-Cadena, Roberto Navarro-López, Adalberto A. Pérez de León, Carlos Guichard-Romero, Estelle Martin, Wendy Tang, Matthias Frank, Monica Borucki, Michael J. Turell, Alex Pauvolid-Corrêa, Mario A. Rodríguez-Pérez, Héctor Ochoa-Díaz-López, Sarah A. Hamer, Gabriel L. Hamer

**Affiliations:** Texas A&M University, College Station, Texas, USA (E. Davila, E. Martin, W. Tang, A. Pauvolid-Corrêa, S.A. Hamer, G.L. Hamer);; Instituto Politécnico Nacional, Reynosa, México (N.A. Fernández-Santos, J.G. Estrada-Franco, L. Wei, J.A. Aguilar-Durán, M. de J. López-López, M.A. Rodríguez-Pérez);; El Colegio de la Frontera Sur, San Cristóbal de Las Casas, México (R. Solís-Hernández, R. García-Miranda, D.D. Velázquez-Ramírez, J. Torres-Romero, H. Ochoa-Díaz-López);; Universidad Autónoma de Chiapas, Tuxtla Gutiérrez, México (S. Arellano Chávez);; Universidad Autónoma de Chiapas, Ocozocoautla de Espinosa, México (R. Cruz-Cadena);; Comisión México-Estados Unidos para la Prevención de la Fiebre Aftosa y Otras Enfermedades Exóticas de los Animales, México City (R. Navarro-López);; US Department of Agriculture Agricultural Research Service Knipling-Bushland Livestock Insects Research Laboratory, Kerrville, Texas, USA (A.A. Pérez de León);; Zoológico Miguel Álvarez del Toro, Tuxtla Gutiérrez (C. Guichard-Romero);; Lawrence Livermore National Laboratory, Livermore, California, USA (M. Frank, M. Borucki);; VectorID LLC, Frederick, Maryland, USA (M.J. Turell)

**Keywords:** flaviviruses, West Nile virus, dengue virus, Zika virus, arboviruses, vector-borne infections, viruses, dogs, pets, Mexico

## Abstract

We tested 294 domestic pet dogs in Mexico for neutralizing antibodies for mosquito-borne flaviviruses. We found high (42.6%) exposure to West Nile virus in Reynosa (northern Mexico) and low (1.2%) exposure in Tuxtla Gutierrez (southern Mexico) but very limited exposure to *Aedes*-borne flaviviruses. Domestic dogs may be useful sentinels for West Nile virus.

Mosquito-transmitted viruses represent substantial health burdens across the Americas. Despite the broad geographic ranges of *Aedes* spp. and *Culex* spp. mosquitoes, the endemicity of human arboviral diseases is incongruent with these vector distributions (*1*,*2*). Animal sentinels may therefore be useful for signaling areas of virus transmission and human risk, especially in resource-poor settings where human diseases may be underreported. Although *Ae. aegypti* mosquitoes have been considered to feed predominantly on humans and *Cx. quinquefasciatus* mosquitoes on birds, our recent work studying host feeding patterns in southern Texas, USA (*3*), and northern Mexico (*4*) has documented substantial feeding on dogs for both species, presenting a novel opportunity to evaluate dogs for possible sentinel surveillance. Because dogs are ubiquitous and share the domestic environment with humans, tracking their exposures might provide evidence for understanding human risk and a sensitive indicator of geographic variation for mosquito-borne disease risk. We aimed to estimate domestic dog exposure to Zika virus (ZIKV), dengue virus 1 (DENV-1) and DENV-2, and West Nile virus (WNV) in northern and southern Mexico based on the presence and quantity of specific neutralizing antibodies as a proxy for human risk.

During 2018–2019, we sampled pet dogs from 3 residential areas in the city of Tuxtla Gutierrez, Chiapas, in southern Mexico and 8 neighborhoods in the city of Reynosa, Tamaulipas, in northern Mexico (Figure). We initially screened serum or plasma samples at a 1:10 dilution, then further tested those that neutralized PFUs by >90% in duplicates at serial 2-fold dilutions that ranged from 1:10 to 1:320 to determine 90% endpoint titers (Appendix). 

**Figure:**
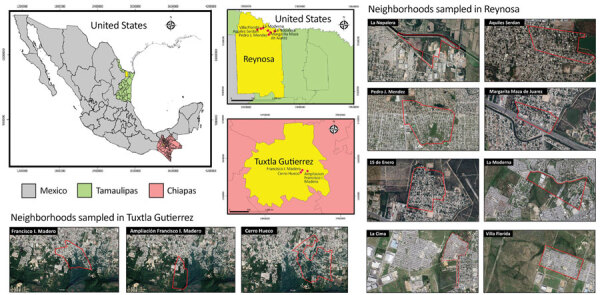
Sampling locations in Tuxtla Gutierrez, Chiapas, and Reynosa, Tamaulipas, Mexico, for study of neutralizing antibodies for mosquito-borne flaviviruses in domestic dogs. Map was created using QGIS 3.18.2 (https://qgis.org/en/site) with public domain map data from Instituto Nacional de Estadística, Geografía e Informatica (National Institute of Statistics, Geography, and Computer Science [INEGI]; https://www.inegi.org.mx/app/mapas) and satellite images from Google Maps (https://www.google.com.mx/maps).

We tested blood samples from 294 pet dogs (predominantly mixed breeds, chihuahuas, and pit bulls). Canine exposure to WNV was widespread, and we found a higher prevalence of neutralizing antibodies to WNV in dogs from Reynosa (72/169, 42.6%) than in those from Tuxtla Gutierrez (1/87, 1.2%; Appendix). In contrast, only 2 (0.7%) dogs from Tuxtla Gutierrez had neutralizing antibodies for ZIKV exposure, showing endpoint titers of 40 and 10. However, the dog with a ZIKV titer of 40 also had a 90% plaque-reduction neutralization test titer of 20 for WNV; we could not screen the dog with a ZIKV titer of 10 for other viruses because of low sample volume. A single dog from Tuxtla Gutierrez had a low titer monotypic reaction for DENV-2, the only evidence of exposure to an *Aedes*-borne flavivirus (Appendix). A sample size analysis indicated that the level of sampling we conducted supports 95% confidence that true prevalence of neutralizing antibodies in these canine populations did not exceed 1% for each of these *Aedes*-borne flaviviruses. 

Our data suggested substantial WNV enzootic activity in Reynosa and corroborated prior observations of high use of dogs as blood meal hosts by *Cx. quinquefasciatus* mosquitoes. Despite detecting neutralizing antibodies for WNV in 42.6% of dogs from Reynosa, the number of reported human WNV cases in Mexico has remained low (*5*), suggesting that transmission occurs among domestic animals but either humans have not been infected or cases have not been reported. Texas has a high number of reported human WNV cases (Texas Department of State Health Services, https://dshs.texas.gov/idcu/disease/arboviral/westNile/#stats). The lower reported numbers of WNV cases in Mexico might be in part because of the high seroprevalence of antibodies for other flaviviruses, which have been shown to protect against severe clinical infection from WNV, thus leading to reduced testing (*6*). Low WNV seroprevalence among dogs in Tuxtla Gutierrez might reflect a larger diversity of vertebrates with lower WNV competence, fed upon by *Culex* mosquitoes in the study area. 

The relative lack of canine exposure to *Aedes*-borne flaviviruses suggests not an absence of these viruses circulating in these communities but that dogs are likely insensitive sentinels of the viruses’ transmission in Mexico. In Chiapas, 7,972 human cases of dengue and 763 cases of Zika had been reported during 2016–2020 (*7*,*8*). Considering the timing of our sampling and the ages of the dogs, we expect that ≈75% of sampled dogs were living in these communities during DENV and ZIKV transmission activity. In the state of Tamaulipas, there were 3,988 human cases of dengue (*7*) and 733 cases of Zika during 2016–2020 (*8*). Given recent quantification that >50% of *Ae. aegypti* in southern Texas and northern Mexico feed on dogs (*3**,**4*), our serologic data suggest that either the probability of virus spillover into dogs is low or that, although dogs are susceptible to infection, neutralizing antibodies developed weakly or waned rapidly (*9*). 

Our study suggests substantial WNV enzootic activity in Reynosa, Mexico and corroborates observations that *Cx. quinquefasciatus* mosquitoes, a primary vector of WNV, use high numbers of dogs for blood meals. Therefore, domestic pet dogs may be useful sentinels of WNV transmission, as previously suggested in other regions (*10*). 

Appendix Additional information about the usefulness of domestic dogs as sentinels for West Nile virus.
