# Supplementary material for: Domestic Dogs as Sentinels for West Nile Virus but not Aedes-borne Flaviviruses, Mexico
Source: Emerg Infect Dis. 2022 May;28(5):1071–4. doi: 10.3201/eid2805.211879 (PMC9045458; doi:10.3201/eid2805.211879)
Supplement: Appendix — Additional information about the usefulness of domestic dogs as sentinels for West Nile virus. [file 21-1879-Techapp-s1.pdf]

Article DOI: https://doi.org/10.3201/eid2805.211879

Domestic Dogs as Sentinels for West Nile Virus but not *Aedes*-borne Flaviviruses, Mexico

Appendix

Description of Study Areas in Northern and Southern Mexico

The city of Reynosa, Tamaulipas, Mexico, with >600,000 residents, covers ≈3,100 km^2 adjacent to the US border across from McAllen, Texas. The municipality of Tuxtla Gutierrez in Chiapas, Mexico covers an area of 412 km^2 and has a population of ≈600,000 residents according to the 2020 population census. The ecologic park there, El Zapotal, contains Zoológico Miguél Álvarez del Toro, a zoo housing fauna endemic to Chiapas.

Methods

Dog Blood Sample Collection

Over 2 different time periods, we collected blood samples from dogs in 2 regions of Mexico. Oversight was performed by the Texas A&M University Institutional Animal Use and Care Committee and the institutional review board of El Colegio de la Frontera Sur.

During December 2018, we sampled pet dogs from 3 residential areas adjacent to El Zapotal, Tuxtla Gutierrez, recruited by door-to-door home visits. During March–October 2019, we collected samples in 8 neighborhoods in Reynosa (Figure). In both study locations, rabies vaccinations for dogs were offered as an incentive for participating pet owners. Owners provided written informed consent and data on dogs' age, sex, and breed. Many owners reported their pets as mongrels when breeds were mixed or unknown.

We collected blood by cephalic, jugular, or medial saphenous venipuncture into tubes containing a clot activator for serum or EDTA (ethylenediaminetetraacetic acid) as an anticoagulant for plasma samples (Becton, Dickinson, and Company; https://www.bd.com). All samples were kept cold until laboratory processing. We spun blood samples and stored aliquots

of serum, clot, whole blood, plasma, and erythrocytes at −80°C for 1–3 mo until shipping them to Texas A&M University for analysis. The Centers for Disease Control and Prevention (CDC) and the US Department of Agriculture issued import permits.

Virus Propagation and Titration

All work involving infectious viruses was performed in a biosafety level 2 facility. We tested all propagates by real-time reverse transcription PCR for ZIKV (*1*), DENV-1 through -4 (*2*), and WNV (*3*) to confirm viral identity. We inoculated virus stocks in T-25 flasks with Vero CCL-81 (American Type Culture Collection; https://www.atcc.org) cultures for virus propagation. When we observed cytopathic effect, we harvested viral suspensions, then centrifuged, filtered, aliquoted, and stored them at −80°C. We then titrated virus strains in Vero cells by plaque assay (*4*).

Plaque Reduction Neutralization Testing

We heat-inactivated serum and plasma samples at 56°C for 30 min and then following standard protocols (*5*) to test them by 90% plaque reduction neutralization testing ($PRNT_{90}$) for their ability to neutralize plaque formation by DENV-1, DENV-2, ZIKV, and WNV. We used the mouse hyperimmune ascitic fluids of all 4 viruses as positive controls.

We initially screened serum and plasma samples at a 1:10 dilution and further tested those that neutralized PFUs by ≥90% in duplicates at serial 2-fold dilutions ranging from 1:10 to 1:320 to determine 90% endpoint titers. We considered serum samples seropositive in a monotypic reaction when a serum dilution in duplicate of ≥1:20 reduced the formation of PFUs ≥90% in only 1 of the 4 flaviviruses tested. We also considered serum samples seropositive in a heterologous reaction when it reduced ≥90% of the formation of plaques of a flavivirus and the reciprocal neutralizing antibody titer was ≥4-fold greater than what was observed for the other 3 tested flaviviruses (*6*). Serum and plasma samples that had $PRNT_{90}$ titers of 10, in either monotypic or heterotypic reactions, or that we could not test for all flaviviruses were considered inconclusive. We considered undetermined those serum samples that presented PRNT titer ≥20 for >1 flavivirus and presented titer difference <4-fold greater for any flavivirus. We considered seronegative those serum samples with PRNT titers <10 for all 4 flaviviruses (*7*).

Statistical Analysis

We calculated seroprevalence for each virus by dividing the total number of confirmed positives by the total number of samples tested for neutralizing antibodies to that particular virus. For WNV testing, some serum samples had insufficient volume to confirm the endpoint titer after screening positive at 1:10. Accordingly, we applied the same percentage of confirmed positive samples to those unconfirmed samples to enable seroprevalence estimation. Because of the large number of dogs in our sample set that tested negative, we performed a post hoc sample size analysis to estimate the maximum number of dogs expected to be seropositive for a virus based on the number of dogs in our study that tested negative. The formula we used was

$$D = [1-(1-a)^{1/n}][N-(n-1)/2],$$

where D is the expected number of seropositive dogs, a is confidence, N is total number of dogs, and n is the subset of dogs tested (*8*).

Results

Of the 256 dogs we tested for WNV, 88 (34.4%) showed antibody titers ≥10. We performed endpoint titers for 83 samples and considered the remaining 5 inconclusive because they had insufficient volume for testing. From the 83 samples fully tested, we confirmed 69 (83.1%) positive for WNV. Applying this 83.1% proportion to the 5 samples that had a titer ≥10 but insufficient volume to confirm a titer ≥20 added 4 WNV-positive dogs, all from Reynosa, to the total. We estimated that 73/256 (28.5%) were positive for WNV antibodies. Among the remaining 183 samples, 11 were negative for all 4 viruses, 2 were inconclusive, and 1 was seropositive for an undetermined flavivirus. Of the 69 confirmed positive, 14 (20.3%) had $PRNT_{90}$ titers ≥320. Among the 69 dogs confirmed positive plus the 4 dogs estimated to be positive for WNV from among the samples with insufficient volume, seroprevalence was significantly higher among dogs from Reynosa 72/169 (42.6%) than dogs from Tuxtla Gutierrez 1/87 (1.2%) ($\chi^2 = 46.41$, $p< 0.001$).

Acknowledgments

We thank the World Reference Center for Emerging Viruses and Arboviruses for providing viral strains of ZIKV (MEX 2–81), DENV-1 (MEX C52), and DENV-2 (INH 125271) and the Division of Vector-Borne Diseases/Arbovirus Diseases Branch of the Centers for Disease Control and Prevention for providing chimeric

WNV (yellow fever virus 17D/WNV Flamingo 383–99) and mouse hyperimmune ascitic fluids (MHIAF) used as positive controls.

References

1. Lanciotti RS, Kosoy OL, Laven JJ, Velez JO, Lambert AJ, Johnson AJ, et al. Genetic and serologic properties of Zika virus associated with an epidemic, Yap State, Micronesia, 2007. Emerg Infect Dis. 2008;14:1232–9. PubMed https://doi.org/10.3201/eid1408.080287

2. Alm E, Lesko B, Lindegren G, Ahlm C, Söderholm S, Falk KI, et al. Universal single-probe RT-PCR assay for diagnosis of dengue virus infections. PLoS Negl Trop Dis. 2014;8:e3416. PubMed https://doi.org/10.1371/journal.pntd.0003416

3. Lanciotti RS, Kerst AJ, Nasci RS, Godsey MS, Mitchell CJ, Savage HM, et al. Rapid detection of west nile virus from human clinical specimens, field-collected mosquitoes, and avian samples by a TaqMan reverse transcriptase-PCR assay. J Clin Microbiol. 2000;38:4066–71. PubMed https://doi.org/10.1128/JCM.38.11.4066-4071.2000

4. Roehrig JT, Hombach J, Barrett AD. Guidelines for plaque-reduction neutralization testing of human antibodies to dengue viruses. Viral Immunol. 2008;21:123–32. PubMed https://doi.org/10.1089/vim.2008.0007

5. Lennette EH, Lennette DA, Lennette ET. Diagnostic procedures for viral, rickettsial, and chlamydial infections. Washington, DC: American Public Health Association; 1995.

6. Calisher CH, Karabatsos N, Dalrymple JM, Shope RE, Porterfield JS, Westaway EG, et al. Antigenic relationships between flaviviruses as determined by cross-neutralization tests with polyclonal antisera. J Gen Virol. 1989;70:37–43. PubMed https://doi.org/10.1099/0022-1317-70-1-37

7. Pauvolid-Corrêa A, Campos Z, Soares R, Nogueira RMR, Komar N. Neutralizing antibodies for orthobunyaviruses in Pantanal, Brazil. PLoS Negl Trop Dis. 2017;11:e0006014. PubMed https://doi.org/10.1371/journal.pntd.0006014

8. Martin S, Meek A, Willeberg P. Veterinary epidemiology—principles and methods; Ames, IA: Iowa State University Press; 1987.

Appendix Table. Demographics and test results of dogs in study of dogs as sentinels of West Nile virus*

Study ID	Age, y/Sex	Breed	Neighborhood	City	ZIKV, titers	DENV-1, titers	DENV-2, titers	WNV, titers	Result
1	Unk/Unk	Unk	REY	REY	<10	<10	<10	40	WNV
6	1.5/F	Mix	LN	REY	<10	<10	<10	80	WNV
13†	3/M	Chihuahua	LN	REY	<10	<10	<10	80	WNV
14†	1/M	Chihuahua	LN	REY	<10	<10	<10	80	WNV
15	3/M	Schnauzer	LN	REY	<10	<10	<10	80	WNV
16	2/M	Poodle	LN	REY	<10	<10	<10	40	WNV
17	3/M	Mix	LN	REY	<10	<10	<10	20	WNV
18	3/F	Mix	LN	REY	<10	<10	<10	80	WNV
21	1/F	Mix	LN	REY	<10	<10	<10	40	WNV
22	1/M	Labrador	LN	REY	<10	<10	<10	80	WNV
34	5/M	Mix	AS	REY	<10	<10	<10	160	WNV
39‡	5/F	Chihuahua	AS	REY	<10	<10	<10	≥320	WNV
42‡	5/M	Chihuahua	AS	REY	<10	<10	<10	40	WNV
45	4/M	Mix	AS	REY	<10	<10	<10	160	WNV
47§	2/F	Mix	PJM	REY	<10	<10	<10	160	WNV
48§	3/F	Mix	PJM	REY	<10	<10	<10	40	WNV
51§	7/F	Mix	PJM	REY	<10	<10	<10	40	WNV
53	1.5/M	Bulldog	PJM	REY	<10	<10	<10	160	WNV
63	3/M	Mix	PJM	REY	<10	<10	<10	≥320	WNV
65	4/F	Pitbull	PJM	REY	<10	<10	<10	80	WNV
66¶	2/F	Mix	MMJ	REY	<10	<10	<10	160	WNV
67¶	2/F	Mix	MMJ	REY	<10	<10	<10	160	WNV
68¶	6/M	Mix	MMJ	REY	<10	<10	<10	40	WNV
69#	Unk/M	Mix	MMJ	REY	<10	<10	<10	160	WNV
70**	3/F	Mix	MMJ	REY	<10	<10	<10	160	WNV
72#	4/F	Mix	MMJ	REY	<10	<10	<10	80	WNV
73**	2/M	Mix	MMJ	REY	<10	<10	<10	80	WNV
74	2/F	Mix	MMJ	REY	<10	<10	<10	≥320	WNV
76††	1.5/M	Pitbull	15DE	REY	<10	<10	<10	160	WNV
78††	4/M	Mix	15DE	REY	<10	<10	<10	80	WNV
79	2/F	Mix	15DE	REY	<10	<10	<10	40	WNV
82	2/M	Mix	15DE	REY	<10	<10	<10	≥320	WNV
83	5/M	Mix	15DE	REY	<10	<10	<10	40	WNV
84	1/M	Mix	15DE	REY	<10	<10	<10	80	WNV
85	9/M	Mix	15DE	REY	<10	<10	<10	160	WNV
92	1/M	Mix	15DE	REY	<10	<10	<10	≥320	WNV
93‡‡	3/M	Mix	15DE	REY	<10	<10	<10	80	WNV
94‡‡	10/M	Mix	15DE	REY	<10	<10	<10	≥320	WNV
102	2/M	Pitbull	LM	REY	<10	<10	<10	40	WNV
104	3/M	Chihuahua	LM	REY	<10	<10	<10	≥320	WNV
108	5/F	Border collie	LM	REY	<10	<10	<10	≥320	WNV
112	2/F	Mix	LM	REY	<10	<10	<10	≥320	WNV
114	4/F	French bulldog	LM	REY	<10	10	<10	<10	Inconclusive
127§§	10/M	Pug	LC	REY	<10	<10	<10	80	WNV
128	3/M	Labrador	LC	REY	<10	<10	<10	10	Inconclusive
129§§	3/F	Pug	LC	REY	<10	<10	<10	≥320	WNV
131	4/F	Mix	LC	REY	<10	<10	<10	40	WNV
137	3/F	Pomeranian	LC	REY	<10	<10	<10	160	WNV
141	2.5/M	Chihuahua	LC	REY	<10	<10	<10	80	WNV
142	6/M	Chihuahua	LC	REY	<10	<10	<10	160	WNV
145	2/M	Chihuahua	LC	REY	<10	10	<10	≥320	WNV
148	5/M	Dachshund	LC	REY	<10	<10	<10	160	WNV
152	2/M	Schnauzer	LC	REY	<10	<10	<10	80	WNV
156	3/F	Mix	LC	REY	<10	<10	<10	80	WNV
157	4/F	Chihuahua	LC	REY	<10	<10	<10	80	WNV
159	4/F	Mix	LC	REY	<10	<10	<10	≥320	WNV
170	10/F	Mix	VF	REY	<10	<10	<10	20	WNV
171	1.5/M	Pitbull	VF	REY	<10	<10	<10	≥320	WNV
172	2/M	Pitbull	VF	REY	<10	<10	<10	20	WNV
178	4/M	Chihuahua	VF	REY	<10	<10	<10	80	WNV
183	2/M	German shepherd	VF	REY	<10	<10	<10	20	WNV
184	7/M	Mix	VF	REY	<10	<10	<10	40	WNV
187	2/M	Mix	VF	REY	<10	<10	<10	80	WNV
194	4/F	Chihuahua	VF	REY	<10	<10	<10	80	WNV
196¶¶	2/F	Mix	VF	REY	<10	<10	<10	160	WNV
197¶¶	14/M	Chihuahua	VF	REY	<10	<10	<10	80	WNV
202	10/F	Mix	VF	REY	<10	<10	<10	160	WNV

Study ID	Age, y/Sex	Breed	Neighborhood	City	ZIKV, titers	DENV-1, titers	DENV-2, titers	WNV, titers	Result
209	5/F	Mix	VF	REY	<10	<10	<10	40	WNV
212	1/F	Chihuahua	VF	REY	<10	<10	<10	160	WNV
214	1/F	Chihuahua	VF	REY	<10	<10	<10	≥320	WNV
241	2/M	Mix	FIM	TGZ	40	<10	<10	20	Und. flavivirus
246	2/F	Pug	FIM	TGZ	10	NT	NT	NT	Inconclusive
278	1/F	Chihuahua	FIM	TGZ	<10	<10	20	<10	DENV-2
313	3/M	Basset hound	CH	TGZ	<10	<10	<10	160	WNV

*15DE, 15 de Enero; AS, Aquiles Serdan; CH, Cerro Hueco; DENV, Dengue virus; FIM, Francisco I. Madero; LC, La Cima; LM, La Moderna; LN, La Nopalera; MMJ, Margarita Maza de Juarez; NT, not tested; PJM, Pedro J. Mendez; REY, Reynosa; TGZ, Tuxtla Gutiérrez; Ukn, unknown; Und, undetermined; VF, Villa Florida; WNV, West Nile virus; ZIKV, Zika virus

†–¶¶ Dogs had same owner.
